# Use of β-caryophyllene to combat bacterial dental plaque formation in dogs

**DOI:** 10.1186/s12917-016-0842-1

**Published:** 2016-10-01

**Authors:** Fábio Alessandro Pieri, Marina Campos de Castro Souza, Ligia Lobato Ramos Vermelho, Marina Lobato Ramos Vermelho, Pedro Griffo Perciano, Fabiano Souza Vargas, Andréa Pacheco Batista Borges, Valdir Florêncio da Veiga-Junior, Maria Aparecida Scatamburlo Moreira

**Affiliations:** 1 Departamento de Ciências Básicas da Vida, Universidade Federal de Juiz de Fora, Rua Israel Pinheiro, 2000, Bairro Universitário, CEP: 35020-220 Governador Valadares, Minas Gerais Brasil; 2 Departamento de Veterinária, Universidade Federal de Viçosa, Av. P.H. Rolfs, s/n, Câmpus UFV, CEP: 36570-000 Viçosa, Minas Gerais Brasil; 3 Instituto de Ciências Exatas, Universidade Federal do Amazonas, Av. Rodrigo Otávio, 6200, Coroado, CEP: 69.080-005 Manaus, Amazonas Brasil

**Keywords:** Antimicrobial, Dogs, Dental plaque, Adhesion, Natural phytochemical

## Abstract

**Background:**

Periodontal disease is a highly prevalent illness that affects many dogs, reaching up to 85 % prevalence in individuals over the age of 4 years. Currently the drug of choice for combating the formation of dental plaque in these animals, the etiologic agent of the disease, is chlorhexidine, which has several side effects reported. Thus, surveys are conducted throughout the world in order to identify potential substitutes for antimicrobial therapy and prevention of periodontal disease. The objective of the work was to evaluate the antimicrobial activity of β-caryophyllene against bacteria from dog’s dental plaque in vitro and in vivo. The minimum inhibitory concentration was evaluated by agar microdilution assay, the induction or inhibition of bacterial adherence by sub-inhibitory concentrations in 96-well plates, and reduction of dental plaque formation in mongrel dogs subjected to topical solution with β-caryophyllene for 15 days.

**Results:**

Results showed minimum inhibitory concentrations above 100 mg/mL for 25 % of the isolates, 100 mg/mL for 3 %, 50 mg/mL for 25 %, 25 mg/mL for 12 %, 12.5 mg/mL for 19 % and 6.25 mg/mL for 16 %. Bacterial adherences of three *Enterococcus* sp., one *Streptococcus* sp., one *Haemophilus* sp., one *Aerococcus* sp., one *Bacillus* sp. and one *Lactococcus* sp. isolates were inhibited by subinhibitory concentration. One *Lactococcus* sp., one *Bacillus* sp. and one *Streptococcus* sp. were stimulated to adhere by concentrations of 0.19, 1.56 and 0.78 mg/mL, respectively. In vivo assay showed reduction in dental plaque formation by β-caryophyllene, with final plaque coverage of 23.3 ± 2.6 % of the total area of the teeth, with significant difference compared with chlorhexidine group (37.5 ± 3.7 % - *p* < 0.05) and negative control group (65.5 ± 2.5 % - *p* < 0.001).

**Conclusions:**

The results showed that β-caryophyllene has antimicrobial activity against the proliferation of dog’s dental plaque-forming bacteria representing a suitable alternative to the use of chlorhexidine in prophylaxis and treatment of periodontal disease of dogs.

## Background

Periodontal disease is a highly prevalent illness that affects many dogs, reaching up to 85 % prevalence in individuals over the age of 4 years [1]. It affects the supporting and protective structures of the teeth and its aetiological agent is the bacterial plaque that develops on the tooth surface, and the immune reaction to infection [2]. Tooth brushing is the most suitable procedure for the prevention of periodontal disease through mechanical removal of dental plaque; however, some alternatives can be employed simultaneously to brushing with the intention of increasing the efficiency of plaque removal. Among these alternatives is the administration of antibacterial substances, which, by preventing the proliferation or adhesion of bacteria to the teeth surface, inhibit the development of periodontal disease [3].

In addition, there is evidence that biofilm formation can be stimulated by certain antimicrobials in sub-inhibitory concentrations, which makes it necessary to investigate a potential stimulus for bacterial adherence by antimicrobial agents, making its use in therapy impossible [4, 5]. Studies have indicated several classes of antibiotics, including tetracyclines, quinopristina-dalfopristin, erythromycin and enrofloxacin, which stimulate the formation of biofilms by *Staphylococcus epidermidis* and *Escherichia coli* [5, 6]. In contrast, the discovery of therapeutic agents that are capable of inhibiting biofilm formation, even in sub-inhibitory concentrations, would be useful for the prevention of periodontal disease by inhibiting the formation of dental plaque [3].

Chlorhexidine is currently the drug of choice for combating dental plaque bacteria, and is usually commercialised at the concentration of 0.12 % [7]. However, the use of this drug has some side effects, such as darkening of the tooth enamel, loss of taste, burning sensation in the oral cavity and ulceration of the oral mucosa, as well as a bitter taste and the fact that it enables the emergence of resistant bacteria [8]. Therefore, there is a need to search for alternatives to this drug for prophylaxis and treatment of periodontal disease [9].

**Fig. 1:**
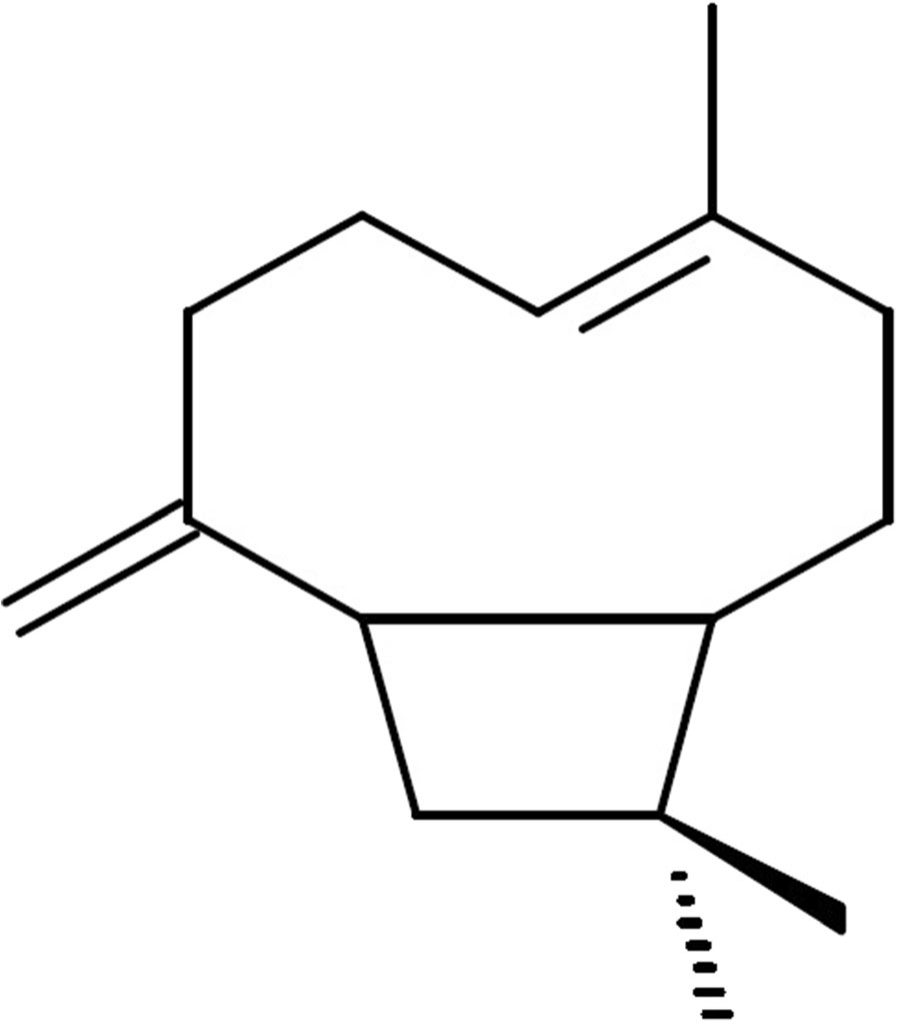
Chemical structure of β-caryophyllene (4,11,11-trimethyl-8-methylene-bicyclo[7.2.0]undec-4-ene)

The β-caryophyllene is a sesquiterpene (C_15_H_24_ – Fig. 1), identified in the CAS (Chemical Abstract Service) under number 87-44-5; this is found in many plant sources, and shows great potential [10]. Studies have shown low toxicity and high applicability, with this substance being used for several purposes, including local anaesthesia [11], anti-inflammatory action [12, 13], antispasmodic action [14], antimicrobial activity [15], anxiolytic [16], and protection against ischemic injury in neurons [17], among others. Several studies to identify antimicrobial activity have been conducted with plants that have high percentages of β-caryophyllene in their composition, presenting positive results for these extracts against several pathogens: *Thymus kotschyanus* [18], *Spiranthera odoratissima* [19], *Lantana* sp. [20], *Vernonia remotiflorae* V. *brasiliana* [21], *Syzygium cumini* [22], and *Lippia gracillis* [23], among others.

Considering the high prevalence of periodontal disease in dogs, the side effects caused by the drug of choice currently to combat microorganisms of dental plaque, and the described antimicrobial potential of β-caryophyllene, the aim of this work was to evaluate the antimicrobial activity of this phytochemical against dental plaque bacteria on dogs both in vitro and in vivo, and evaluate its ability to interfere in the adherence of bacterial plaque isolates.

## Methods

### Bacterial isolates

Thirty two bacterial isolates were used from ten different genera, which were obtained from dental plaque of mongrel dogs aged between 1 and 2 years, fed with dry feed ad libitum. The number of isolates within each genus was selected following the same percentage that they represented within the total isolates obtained from dogs in the study performed by Pieri [24]: six *Streptococcus* sp., six *Staphylococcus* sp., six *Enterococcus* sp., three *Lactobacillus* sp., three *Leuconostoc* sp. two *Actinomyces* sp., two *Bacillus* sp., two *Lactococcus* sp., one *Aerococcus* sp., and one *Haemophilus* sp.

### Minimum inhibitory concentration (MIC)

The in vitro assay was performed by adaptation of the agar macrodilution technique [25], which was modified for agar microdilution performed in 96-well microtitre plates. The β-caryophyllene (≥98.5 %, Sigma-Aldrich, Saint Louis, MO, USA) was incorporated within agar brain heart infusion (BHI), in serial dilutions of logarithmic base 2, in concentrations ranging between 100 and 6.25 mg/mL. As a positive control, chlorhexidine digluconate was used at concentrations between 20 and 0.015 mg/mL, and bacterial growth control was performed with agar BHI without active principle.

The assay was prepared in triplicate for each isolate, filling corresponding wells with 150 μL of culture medium with different concentrations of β-caryophyllene, 150 μL with chlorhexidine or 150 μL of unsupplemented culture medium. Each well except the last row (non-inoculated as a control of culture medium sterility) was inoculated with 3 μL of the adjusted 3 × 10^7^ UFC/mL and the plates were incubated for 24 h at 37 °C. After incubation, the bacterial growth was evidenced by the addition of 50 μL resazurin solution at 0.01 % as a colorimetric indicator of oxireduction to characterise cell viability. Interpretation of the results was based on the conversion of resazurin in resorufin by microbial metabolism, showing a change of the dye colour from blue to pink when microbial growth occurred. The MIC was based on the lowest concentration of β-caryophyllene and chlorhexidine in which the dye remained blue, indicating the absence of microbial growth.

### Stimulation/inhibition of bacterial adherence

The quantification of inhibition or stimulation of the bacterial adherence was performed by a microtiter-plate test for quantification biofilm formation according [26], with modifications performed by [27]. Here, 24 bacterial isolates from dog dental plaque of the following genera were tested: *Actinomyces* sp. (2), *Aerococcus* sp. (1), *Bacillus* sp. (1), *Enterococcus* sp. (6), *Haemophilus* sp. (1), *Lactobacillus* sp. (1), *Lactococcus* sp. (2), *Leuconostoc* sp. (2), *Staphylococcus* sp. (3) and *Streptococcus* sp. (5). From the MIC of β-caryophyllene against each isolate, six descending serial dilutions of logarithmic base 2 were used (final concentrations). The isolates were reactivated in BHI incubated at 37 °C for 24 h and then adjusted to 0.5 McFarland scale (1.5 × 10^8^). For this, 230 μL of each isolate culture was added to the wells of 96 well microtitre plates, and 70 μL of test solution was added to assess the final six different concentrations of each isolate, in triplicate. The positive control of bacterial adherence for each isolate consisted of the addition of 230 μL of adjusted bacterial culture and 70 μL of sterile BHI broth, and the negative control of bacterial adherence was prepared with 300 μL of sterile BHI broth. The plates were incubated at 37 °C for 24 h. After this period, the content was discarded from the plates and these were washed three times with distilled water to remove non-adhered bacteria. Then, 250 μL of methanol was added to each well, which was allowed to stand for 15 min. The methanol was discarded, and the plate was dried for 2 min in laminar flow, following staining with 250 μL of 1 % crystal violet per well for 10 min. The dye was removed from the plate with tap water and then 250 μL of 33 % glacial acetic acid was added. The optical density of each well was measured by a microplate spectrophotometer. With the aid of statistical software Prism 5 (GraphPad Software Inc., La Jolla-CA, USA) the results were analysed by one-way ANOVA comparing treatments to the positive control using the Dunnett’s test.

### Inhibition of dental plaque formation in dogs

Here, 18 healthy mongrel dogs, aged between 2 and 8 years old, divided randomly in males and females, were divided into three groups: negative control group (treat solution: tween 80, butylated hydroxytoluene, sodium benzoate and distilled water); positive control group (negative control solution with 0.12 % chlorhexidine gluconate – commercial concentration of this drug) and test group (negative control solution with 50 mg/mL of β-caryophyllene added). The dogs were kept during the experimental period, two by two, in masonry kennels (1.50 m × 3.00 m).

At the beginning of the experiment, all animals were subjected to a dental cleaning with dental ultrasound (Profi II AS ceramic, Dabi Atlante, Ribeirão Preto, Brazil) and curettage, for the total exclusion of dental plaque, confirmed with a disclosing solution (0,5 % basic fuchsin solution). The animals in each group were treated with the respective solution twice daily for 15 days. All were fed the same dry food and water ad libitum. At the end of the experimental period, the bacterial dental plaque formed on teeth vestibular surfaces, of canines and pre molars, were observed using 0.5 % basic fuchsin for bacterial identification. These regions were photographed, with digital camera positioned perpendicularly to the imaged surface, distant 30 cm from the animals, and the images were subjected to analysis in graphic software ImageJ 1.44p (National Institute of Health, Bethesda-MD, USA) to obtain the percentage of total tooth surface area with the presence of dental plaque. Vestibular areas of canine and all premolar teeth of each dog were considered for this analysis. Data were subjected to one-way ANOVA and the parametric Tukey test was used to compare treatment means using the Prism 5 software. For this study, *P* < 0.05 was considered significant.

## Results and discussion

### Minimum inhibitory concentration

**Fig. 2:**
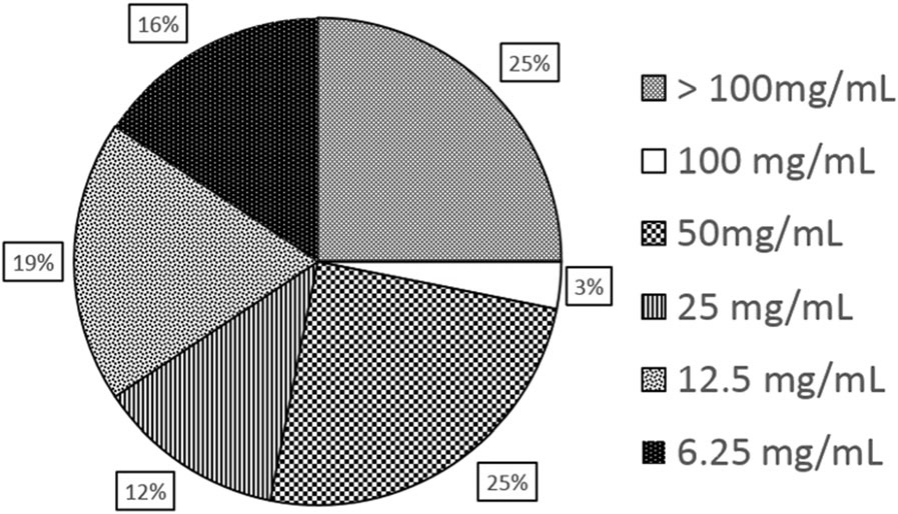
Distribution of sensitivity of 32 dental plaque bacterial isolates obtained from dogs classified based on minimum inhibitory concentrations of β-caryophyllene

The results of the MIC test for β-caryophyllene and chlorhexidine against dental plaque bacterial isolates are shown in Table 1. Overall, 75 % (24/32) of the tested isolates were sensitive to β-caryophyllene at concentrations up to 100 mg/mL. The results of the inhibition of *Streptococcus* sp. should be highlighted, as streptococci are described as the most important in the initial adhesion of dental plaque in humans [28], and all isolates were inhibited by β-caryophyllene. This fact suggests that this compound could also be a potential alternative for dental plaque inhibition in humans, after further studies using strains isolated from human samples. The MIC for 50 % (3/6) of isolates of *Streptococcus* sp. was ≤ 6.25 mg/mL, for 33.3 % (2/6) was between 6.25 and 12.5 mg/mL, and for 16.7 % (1/6) was between 25 and 50 mg/mL. The isolates showed sensitivity to the tested substance with respect to the MIC profile, as shown in Fig. 2.

**Table 1:** Minimum inhibitory concentrations of β-caryophyllene and chlorhexidine against 32 bacterial isolates obtained from dog dental plaque

Code	Bacterial genus	β-caryophyllene	Chlorhexidine
HQ717206	*Actinomyces* sp.	25 (mg/mL)	≤0.015 mg/mL
HQ717208	*Actinomyces* sp.	12.5 (mg/mL)	≤0.015 mg/mL
HQ717237	*Aerococcus* sp.	25 (mg/mL)	≤0.015 mg/mL
HQ717211	*Bacillus* sp.	50 (mg/mL)	≤0.015 mg/mL
HQ717289	*Bacillus* sp*.*	>100 (mg/mL)	≤0.015 mg/mL
HQ717176	*Enterococcus* sp.	50 (mg/mL)	≤0.015 mg/mL
HQ717205	*Enterococcus* sp.	50 (mg/mL)	≤0.015 mg/mL
HQ717227	*Enterococcus* sp.	50 (mg/mL)	≤0.015 mg/mL
HQ717268	*Enterococcus* sp.	12,5 (mg/mL)	≤0.015 mg/mL
HQ717302	*Enterococcus* sp*.*	12.5 (mg/mL)	≤0.015 mg/mL
HQ717350	*Enterococcus* sp*.*	25 (mg/mL)	≤0.015 mg/mL
HQ717319	*Haemophilus* sp.	100 (mg/mL)	≤0.015 mg/mL
HQ717266	*Lactobacillus* sp.	12.5 (mg/mL)	≤0.015 mg/mL
HQ717270	*Lactobacillus* sp.	50 (mg/mL)	≤0.015 mg/mL
HQ717278	*Lactobacillus* sp.	50 (mg/mL)	≤0.015 mg/mL
HQ717330	*Lactococcus* sp*.*	6.25 (mg/mL)	≤0.015 mg/mL
HQ717335	*Lactococcus* sp.	6.25 (mg/mL)	≤0.015 mg/mL
HQ717296	*Leuconostoc* sp.	50 (mg/mL)	≤0.015 mg/mL
HQ717308	*Leuconostoc* sp.	>100 (mg/mL)	≤0.015 mg/mL
HQ717331	*Leuconostoc* sp.	>100 (mg/mL)	≤0.015 mg/mL
HQ717182	*Staphylococcus* sp.	25 (mg/mL)	≤0.015 mg/mL
HQ717223	*Staphylococcus* sp.	>100 (mg/mL)	≤0.015 mg/mL
HQ717224	*Staphylococcus* sp.	>100 (mg/mL)	≤0.015 mg/mL
HQ717232	*Staphylococcus* sp.	>100 (mg/mL)	≤0.015 mg/mL
HQ717306	*Staphylococcus*sp.	>100 (mg/mL)	≤0.015 mg/mL
HQ717309	*Staphylococcus* sp.	>100 (mg/mL)	≤0.015 mg/mL
HQ717228	*Streptococcus* sp.	50 (mg/mL)	≤0.015 mg/mL
HQ717229	*Streptococcus* sp.	6.25 (mg/mL)	≤0.015 mg/mL
HQ717242	*Streptococcus* sp.	6.25 (mg/mL)	≤0.015 mg/mL
HQ717243	*Streptococcus* sp.	6.25 (mg/mL)	≤0.015 mg/mL
HQ717249	*Streptococcus* sp.	12.5 (mg/mL)	≤0.015 mg/mL
HQ717305	*Streptococcus* sp.	12.5 (mg/mL)	≤0.015 mg/mL

Codes presented are the respective numbers of deposit of the 16S rRNA sequences of each isolate in GenBank

Relevant findings of antimicrobial activity by β-caryophyllene was described by Huang et al. [15], who found that strains of *Arabidopsis thaliana* flowers that did not have the compound in their chemical composition showed greater growth of bacteria on their stigmas compared with the wild type that showed β-caryophyllene in its composition.

In vitro studies showed natural products that are present as the major compound β-caryophyllene, with significant antimicrobial activity, suggesting that the substance possibly participated in this activity. Da Costa et al. [20] studied the antibacterial activity of extract of *Lantana camara*, composed 31.5 % byβ-caryophyllene. The results showed significant antimicrobial activity, especially against *Proteus vulgaris* (ATCC 13315) and *Escherichia coli* (ATCC 25922). Maia et al. [21] presented the antibacterial activity of the essential oils of *Vernonia remotiflora* and *V. braziliana*, both with more than 40 % β-caryophyllene, with a broad spectrum of antibacterial activity, inhibiting the growth of several tested Gram-negative and Gram-positive bacteria, including *S. aureus* and *Pseudomonas aeruginosa.*

Ghosh et al. [29] evaluated the activity of essential oils of *Alpinia nigra* (47.7 to 49 % of β-caryophyllene) against Gram-positive and Gram-negative bacteria. The lowest MIC was found against *Yersinia enterocolitica* (1.56 μL/mL). In the present study, *Staphylococcus* isolates had relatively high MICs (one was 25 mg/mL and five were resistant up to 100 mg/mL) compared to the isolate of *S. aureus* in the study of Ghosh et al. [29], which showed MICs ranging between 3.12 and 6.25 μL/mL depending on the essential oil of *A. nigra* analysed. This difference in susceptibility of bacteria of the same genus may have occurred by synergistic action of β-caryophyllene with other substances present in the tested oil [30], or due to the intrinsic resistance of wild isolates of the present study to this phytotherapic was higher when compared to that strain tested by Ghosh et al. [29].

Souza et al. [30] investigated the antibacterial activity of β-caryophyllene against cariogenic bacteria in humans, *Streptococcus mutans*, *S. mitis*, *S. sobrinus*, *S. sanguinis*, and *Lactobacillus casei*, and showed MICs of 200, 150, 180, 200 and 150 μg/mL, respectively. These concentrations were lower than those found in the present study, indicating a higher susceptibility of bacteria in human dental plaque than those isolated from dogs, making it an even more promising compound for use in dental therapy in humans.

Corroborating this promising employment in human dental therapy, Mussi [31] conducted tests of MIC with one oil from *Copaifera officinalis* (with 50.78 % β-caryophyllene) against bacteria that cause severe periodontal disease in humans: *Fusobacterium nucleatum* and *Porphyromonas gingivalis*. The solution of copaiba was active against *F. nucleatum* from the concentration 100 μL/mL, while the MIC against *P. gingivalis* was 2 μL/mL. However, for *P. gingivalis* the nature of the antimicrobial activity was bacteriostatic at all concentrations tested, while for *F. nucleatum* the MIC was also the minimum bactericidal concentration (MBC).

### Stimulation/inhibition of bacterial adherence

Table 2 lists the isolates that showed some inhibition or stimulation in its adherence capability by some sub-inhibitory concentration of β-caryophyllene, with statistical significance (*P* < 0.05) when compared with the control. Within the 24 isolates tested, eight (33.33 %) showed an altered ability to form biofilms enough to be detected by statistical analysis. An isolate of *Lactococcus* sp. suffered inhibition of its adherence by concentrations of 1.56 mg/mL % and 3.12 mg/mL (*P* < 0.05), while concerning the challenge with 0.19 mg/mL of β-caryophyllene, the activity was the stimulus to the adherence of the same isolate (*P* < 0.05). One *Streptococcus* sp. and one *Bacillus* sp. presented similar results, being inhibited to adhere by some concentrations and stimulated by other one (Table 2).

**Table 2:** Stimulation/Inhibition of bacterial adherence in microtiter plates by subinhibitory concentrations of β-caryophyllene

Code	Bacterial genus	Subinhibitory concentration (mg/mL)	Action on bacterial adherence	Control OD mean (SE)	Treated OD mean (SE)
HQ717249	*Streptococcus* sp.	6.25	Inhibition	0.306 (0.018)	0.093 (0.011)
		3.12	Inhibition	0.306 (0.018)	0.106 (0.009)
		1.56	Inhibition	0.306 (0.018)	0.117 (0.013)
		0.78	Stimulus	0.306 (0.018)	0.578 (0.021)
HQ717319	*Haemophilus* sp.	50.00	Inhibition	0.125 (0.001)	0.076 (0.001)
		25.00	Inhibition	0.125 (0.001)	0.071 (0.001)
		12.50	Inhibition	0.125 (0.001)	0.087 (0.004)
		6.25	Inhibition	0.125 (0.001)	0.089 (0.003)
HQ717330	*Lactococcus* sp.	3.12	Inhibition	1.072 (0.050)	0.203 (0.065)
		1.56	Inhibition	1.072 (0.050)	0.458 (0.080)
		0.19	Stimulus	1.072 (0.050)	1.590 (0.157)
HQ717350	*Enterococcus sp.*	12.50	Inhibition	0.209 (0.005)	0.076 (0.001)
HQ717176	*Enterococcus sp.*	25.00	Inhibition	0.114 (0.003)	0.075 (0.001)
		12.50	Inhibition	0.114 (0.003)	0.073 (0.005)
		6.25	Inhibition	0.114 (0.003)	0.065 (0.001)
		1.56	Inhibition	0.114 (0.003)	0.073 (0.003)
HQ717227	*Enterococcus* sp.	25.00	Inhibition	0.676 (0.061)	0.094 (0.017)
		12.50	Inhibition	0.676 (0.061)	0.075 (0.007)
		6.25	Inhibition	0.676 (0.061)	0.068 (0,001)
		3.12	Inhibition	0.676 (0.061)	0.083 (0.005)
		1.56	Inhibition	0.676 (0.061)	0.122 (0.017)
HQ717211	*Bacillus* sp.	25.00	Inhibition	0.398 (0.008)	0.088 (0.013)
		12.50	Inhibition	0.398 (0.008)	0.077 (0.009)
		6.25	Inhibition	0.398 (0.008)	0.112 (0.031)
		3.12	Inhibition	0.398 (0.008)	0.168 (0.019)
		1.56	Stimulus	0.398 (0.008)	0.517 (0.055)
HQ717237	*Aerococcus* sp.	12.50	Inhibition	0.119 (0.005)	0.082 (0.002)
		1.56	Inhibition	0.119 (0.005)	0.071 (0.001)

Codes presented are the respective numbers of deposit of the 16S rRNA sequences of each isolate in GenBank. Means (±SE) of optical density are related to measurement under 550 nm absorbance (OD_550_). All of the presented results showed a significant difference between the test treatment and the control of isolates natural adherence without any treatment (*P* < 0.05)

It can be seen in Table 2 that the eight isolates that showed statistical differences in the adherence to microtitre plate orifices were from six different genera, *Streptococcus* sp. (1/5), *Haemophilus* sp. (1/1), *Aerococcus* sp. (1/1), *Lactococcus* sp. (1/2), *Bacillus* sp. (1/1) and *Enterococcus* sp. (3/6). As more than one isolate of each genus were tested for *Streptococcus*, *Enterococcus* and *Lactococcus*, it can be stated, at least to these genera, that the susceptibility to changes in adherence capability is characterised in strain level. This fact was presented before [6], with different *E. coli* strains presenting different interference levels in biofilm formation when challenged by enrofloxacin.

Pieri et al. [3] studied the effects of *Copaifera officinalis* oil, which is described in the work of Mussi [31] as containing more than 50 % β-caryophyllene, on the adhesion of the bacteria *Streptococcus mutans*, the main involved in the initial formation of dental plaque in humans. Analysis of the inhibition of adherence assays showed superiority of the test group compared with the negative control and positive control with chlorhexidine. In the present study, an isolate of the same genus had its adhesiveness inhibited by sub-inhibitory concentrations of β-caryophyllene between 1.56 and 6.25 mg/mL and stimulated to adhere at 0.78 mg/mL. However, other isolates of *Streptococcus* sp. were also tested, but presented no significant change in their ability to adhere, which supports the individuality of each strain with respect to variation in adherence capability by the tested compound.

Mussi [31] conducted tests to evaluate the co-aggregation and self-aggregation (processes related to microbial adherence in dental plaque of the advanced periodontal disease) by the periodontal pathogens *Fusobacterium nucleatum* and *Porphyromonas gingivalis* challenged with sub-inhibitory concentrations of *Copaifera officinalis* oil, composed of 50.78 % of β-caryophyllene. When *F. nucleatum* was treated, a reduction in the self-aggregation process was observed when compared to untreated cells. *P. gingivalis* was not able to self-aggregate under the tested conditions. The solution of *C. officinalis* tested also inhibited co-aggregation between these two bacterial species, which is an important event in the progression of periodontal disease in humans, again demonstrating the great employment potential of β-caryophyllene in human dentistry.

It’s significant to highlight that in the present work we used aerobes as target strains. This kind of bacteria are not involved in the etiology of periodontal disease, but are critical to early stages in development of dental biofilm, creating from its installation, a favorable environment for the development of anaerobes, which actively participate in the development of the disease [32]. Thus the results of this study point to a relevant activity of β-caryophyllene on aerobes, presenting relevant potential in use to combat dental plaque formation in its early stages. The use in combating plaque formation could promote a prevention of the disease through a reduction of the environmental conditions that promote the development of periodontal pathogens in the biofilm. However, further studies should be conducted evaluating the direct antimicrobial effect on the anaerobic periodontal pathogens, which may point, beyond the preventive potential, the possibility of use β-caryophyllene in the treatment of disease.

### Inhibition of dental plaque formation in dogs

**Fig. 3:**
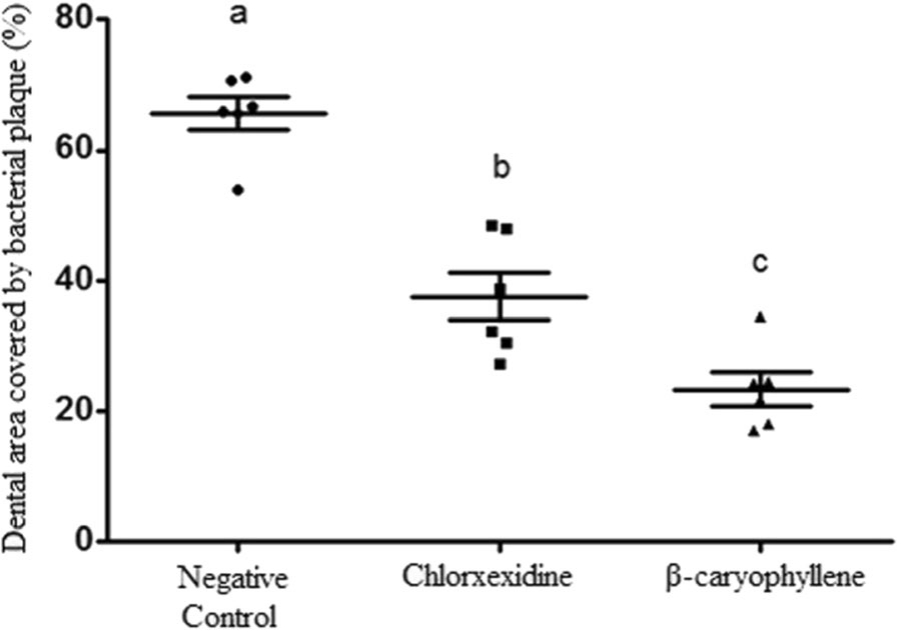
Dental plaque coverage on teeth surfaces in eighteen dogs (six per group), after 15 days of treatment with different solutions. Results are expressed by percentage of total area of vestibular surface of canines and premolars teeth that was covered by plaque. Results were submitted to ANOVA and means were compared by tukey test. Different letters (**a**, **b** and **c**) indicate statistical significant difference (*P* <0.05). central bars: mean; marginal bars: standard error

The results obtained in the in vivo assay are shown in Fig. 3. The results indicated the coverage area of teeth with plaque (mean ± SE) to be 23.3 ± 2.6 % for β-caryophyllene test solution, 37.5 ± 3.7 % for the positive control solution and 65.56 ± 2.5 % coverage for the negative control solution. The test and positive control groups were statistically lower on average than the negative control (*P* < 0.001) dental plaque presence and were different (*P* < 0.05) with regard to inhibiting dental plaque formation, with better results for β-caryophyllene. These results indicate that β-caryophyllene is a potential natural alternative to the use of chlorhexidine in reducing dental plaque in dogs, resulting in a patent application of pharmaceutical formulations containing β-caryophyllene for use in the treatment and prophylaxis of canine periodontal disease [33].

A similar assay was performed by Pieri et al. [3] using one *Copaifera officinalis* oil with good results in the inhibition of dental plaque formation when compared to negative control. In their work, chlorhexidine reduced the formation of dental plaque to a final coverage of 28.5 % of the analysed area of teeth, while in the present work, the group treated with the same substance presented 37.5 % plaque coverage. This difference could be happen due to different susceptibility to chlorhexidine of the dogs’ dental microbiota in the different works, or the different treatment periods, which was 8 days in the previous work and 15 days in the current study. However, it is interesting to highlight that the level of final plaque coverage of groups treated with *C. officinalis* [3] and β-caryophyllene (present work) were very close. As this oil is frequently associated with high concentrations of β-caryophyllene, the activity of *C. officinalis* oil used in that work against dogs’ dental plaque [3] could be due to the presence of this compound.

## Conclusions

With the data obtained it can be concluded that β-caryophyllene has antimicrobial activity against dental plaque bacteria of dogs, and this activity is reflected in the reduction of this on the teeth surfaces of dogs. Therefore, after other clinical trials with a larger number of dogs and different breeds, β-caryophyllene could be indicated as an alternative to the use of chlorhexidine in the treatment and prophylaxis of periodontal disease in these animals.

## Abbreviations

ANOVA: Analysis of variance
ATCC: American type culture collection
BHI: Brain heart infusion
CEUA: Ethics Committee on Animal Use
COBEA: Brazilian College for Animal Experimentation
MBC: Minimum bactericidal concentration
MIC: Minimum inhibitory concentration
SE: Standard error
